# Editorial: Bladder Cancer – A Cinderella Cancer: Advances and Remaining Research Questions

**DOI:** 10.3389/fonc.2020.01749

**Published:** 2020-09-04

**Authors:** Mieke Van Hemelrijck, Prashant Patel, Kent W. Mouw

**Affiliations:** ^1^School of Cancer and Pharmaceutical Sciences, Translational Oncology and Urology Research (TOUR), King's College London, London, United Kingdom; ^2^Institute of Cancer and Genomic Sciences, University of Birmingham, Birmingham, United Kingdom; ^3^Department of Radiation Oncology, Dana–Farber Cancer Institute/Brigham & Women's Hospital and Harvard Medical School, Boston, MA, United States

**Keywords:** bladder cancer, diagnostics, personalization, surveillance, prognostic

Bladder cancer is the 4th most common male cancer and 9th most common female malignancy. Despite its high incidence and prevalence, clinical outcomes have been largely static over the past 25 years. In 2010, bladder cancer was the 9th most expensive cancer in the USA with cumulative costs of 4 billion US dollars or 3.2% of all cancer-related care. A potential and significant contributing factor for the relative lack of improvement in the static mortality rate of BC is the small investment in bladder cancer research. In the UK, prostate cancer research is supported with over £26,458,355 in funding and £561 are spent per new patient. However, research spent on bladder cancer was only £3,886,966 with £382 spent per new patient. A similar funding discrepancy is seen in the USA. In addition to the lack of research funding, bladder cancer has the highest lifetime treatment costs per patient of all cancers. This historical lack of funding means there are now many important unanswered research questions, making prioritization very challenging (1).

Our understanding of factors influencing BC risk and development is improving, but variability across ethnicities has been observed. Wu et al. performed a three-stage case-control study including 3,399 BC patients and identified a novel rare coding variant that increased BC risk in Han Chinese. This may inform future development of novel targeted agents in BC development and progression.

However, it is important to note that the diagnostic pathway of BC is not straightforward due to the high potential of delayed presentation and misinterpretation of potential symptoms like haematuria. Nevertheless, urine biopsies have been suggested to be “liquid gold” as they are less invasive than tumor tissue biopsies and can be an abundant source of tumor-derived material (Satyal et al.). The molecular detection of mutations in urine DNA requires a sensitive and accurate method of analysis—so despite its potential for diagnosis, prognosis, and monitoring of tumor evaluation, significant challenges are apparent and research is still evolving. It is therefore exciting to note that Lipunova et al. externally replicated an algorithm for BC prognosis based on urinary polymorphisms using data from the UK Biobank.

Many other initiatives are ongoing in the area of BC risk stratification to evaluate and refine both new markers as well as existing procedures, such as performing restaging transurethral resection (i.e., repeat TUR) (Calò et al.) and evaluating pre-treatment neutrophil-lymphocyte ratios (Suh et al.). In addition, specific gene expression profiles have been associated with poor prognosis in patients with non-muscle invasive BC (NMIBC) (Chen et al.). By investigating the relation between circular RNAs (circRNAs) and tumor grade in a cohort of NMIBC, another study showed that the assessment of expression levels of circRNAs may provide an additional layer of information for patient stratification (Goel et al.). Apart from providing new avenues for precision medicine, these studies also help us understand how we can bring various observations together—including the rapidly emerging field of immunotherapy and surveillance for BC (Joseph and Enting).

This rapid expansion of diagnostic and prognostic factors for BC also requires strategic thinking for the management of big data. Zhang et al. developed an online consensus Survival tool for bladder cancer (OSblca) to analyze the prognostic value of specific gene expression patterns by integrating genetic and clinical data from 1,075 BC patients. Another initiative providing opportunities for real world evidence research is data harmonization across various hospitals to provide a prospective database with detailed baseline information as well as clinical follow-up—such a database for NMIBC was set-up in Belgium in 2013 (Akand et al.).

Finally, whilst it is encouraging to see a significant increase in BC research, all researchers should be encouraged to engage with BC patients and their carers when designing and conducting research studies as to ensure the best impact on their care (MacLennan and MacLennan).

## Author Contributions

All authors listed have made a substantial, direct and intellectual contribution to the work, and approved it for publication.

## Conflict of Interest

The authors declare that the research was conducted in the absence of any commercial or financial relationships that could be construed as a potential conflict of interest.
